# Verbal fluency tests assess global cognitive status but have limited diagnostic differentiation: evidence from a large-scale examination of six neurodegenerative diseases

**DOI:** 10.1093/braincomms/fcad042

**Published:** 2023-02-21

**Authors:** Shalom K Henderson, Katie A Peterson, Karalyn Patterson, Matthew A Lambon Ralph, James B Rowe

**Affiliations:** MRC Cognition and Brain Sciences Unit, University of Cambridge, Cambridge CB2 7EF, UK; Department of Clinical Neurosciences, University of Cambridge, Cambridge CB2 7EF, UK; Department of Clinical Neurosciences, University of Cambridge, Cambridge CB2 7EF, UK; Cambridge University Hospitals NHS Foundation Trust, Cambridge CB2 7EF, UK; MRC Cognition and Brain Sciences Unit, University of Cambridge, Cambridge CB2 7EF, UK; Department of Clinical Neurosciences, University of Cambridge, Cambridge CB2 7EF, UK; Cambridge University Hospitals NHS Foundation Trust, Cambridge CB2 7EF, UK; MRC Cognition and Brain Sciences Unit, University of Cambridge, Cambridge CB2 7EF, UK; Cambridge University Hospitals NHS Foundation Trust, Cambridge CB2 7EF, UK; MRC Cognition and Brain Sciences Unit, University of Cambridge, Cambridge CB2 7EF, UK; Department of Clinical Neurosciences, University of Cambridge, Cambridge CB2 7EF, UK; Cambridge University Hospitals NHS Foundation Trust, Cambridge CB2 7EF, UK

**Keywords:** Alzheimer’s disease, frontotemporal dementia, Parkinson-plus disorders, primary progressive aphasia, verbal fluency

## Abstract

Verbal fluency is widely used as a clinical test, but its utility in differentiating between neurodegenerative dementias and progressive aphasias, and from healthy controls, remains unclear. We assessed whether various measures of fluency performance could differentiate between Alzheimer’s disease, behavioural variant of frontotemporal dementia, non-fluent and semantic variants of primary progressive aphasia, progressive supranuclear palsy, corticobasal syndrome and healthy controls. Category and letter fluency tasks were administered to 33 controls and 139 patients at their baseline clinical visit. We assessed group differences for total number of words produced, psycholinguistic word properties and associations between production order and exemplar psycholinguistic properties. Receiver operating characteristic curves determined which measure could best discriminate patient groups and controls. The total word count distinguished controls from all patient groups, but neither this measure nor the word properties differentiated the patient groups. Receiver operating characteristic curves revealed that, when comparing controls to patients, the strongest discriminators were total word count followed by word frequency. Word frequency was the strongest discriminator for semantic variant of primary progressive aphasia versus other groups. Fluency word counts were associated with global severity as measured by Addenbrooke’s Cognitive Examination Revised. Verbal fluency is an efficient test for assessing global brain–cognitive health but has limited utility in differentiating between cognitively and anatomically disparate patient groups. This outcome is consistent with the fact that verbal fluency requires many different aspects of higher cognition and language.

## Teaser

Henderson *et al*. report that verbal fluency is an efficient and sensitive test for assessing global brain–cognitive health but is limited in differentiating between different types of dementia and primary progressive aphasia.

## Graphical Abstract

**Graphical Abstract:**
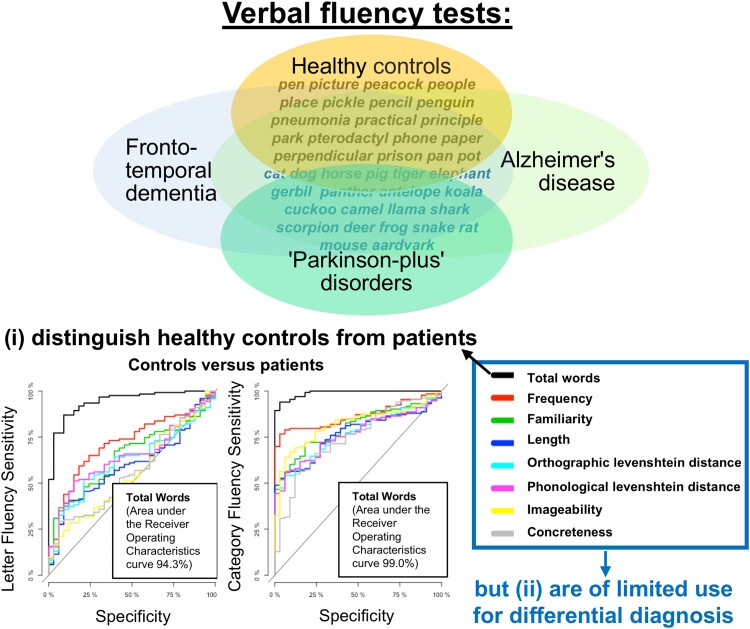

## Introduction

Beyond brief clinician-rated global assessment instruments such as the Clinical Dementia Rating (CDR) scale, verbal fluency tests are one of the most widely used assessments in clinical and research settings. They are quick and easy to administer and score, require no assessment equipment, can differentiate between healthy populations and those with neurodegenerative disease and are sensitive to cognitive or language decline.^1,2^ Verbal fluency tests assess an individual’s ability to generate words from a specified letter of the alphabet (e.g. F, A and S) or a semantic category (e.g. animals and fruits). Difficulty or errors may arise from impairments in one or more aspects of cognition including attention, working memory, semantic memory, executive functioning and language. Verbal fluency deficits have been identified in Alzheimer’s disease, frontotemporal dementia (FTD), vascular dementia, dementia with Lewy bodies, progressive supranuclear palsy (PSP) and other parkinsonian disorders.^3–5^ Typically, previous studies have focussed on one diagnostic group to explore the qualitative and quantitative changes (e.g. total number of words and amount of clustering and switching) in the patients’ performance and pattern of words produced.^6–9^ Whereas the total word count may be an indicator of cognitive impairment, it remains unclear how well verbal fluency tests can differentiate all patient types from healthy controls or between patient groups.^5,6^ In addition, differential impairment between the two fluency tasks, namely a greater impairment for category relative to letter fluency, has been proposed to be a signature of specific syndromes such as semantic dementia (SD) and Alzheimer’s disease.^9–13^ However, there are variations in how this disparity has been calculated, particularly how it relates to the differential performance observed in healthy controls, and investigated across diagnostic groups. The current study used a large-scale transdiagnostic approach to examine letter and category fluency performance assessed at first clinical visit in six different clinical groups representing various cortical and subcortical neurodegenerative disorders including primary progressive aphasias (PPA). We asked some simple but clinically important questions: (i) how well does verbal fluency performance distinguish each group from healthy controls at this first clinical visit; (ii) do these tests contribute to differential diagnosis between patient groups; and (ii) can differential diagnosis be improved if we move beyond total word count to a more detailed analysis of the characteristics of the words produced?

The lexico-semantic features of words produced in tests of verbal fluency (e.g. frequency, imageability and age of acquisition) are less studied than other measures.^14^ In semantic variant of primary progressive aphasia (svPPA)/SD, connected speech production and naming show a relative overuse of words that are more frequent, more abstract (i.e. ‘lighter’ nouns and verbs) and acquired earlier.^15–17^ This pattern is reported to a lesser extent in Alzheimer’s disease with an over-reliance on words that are more frequent and acquired earlier.^18–22^ Although verbal fluency is severely reduced in PSP and corticobasal syndrome (CBS), the psycholinguistic properties of the words have not been investigated in detail: it has been proposed that PSP leads to production of few words of relatively low frequency.^9,23,24^ With the exception of reduced word length observed in non-fluent variant PPA (nfvPPA),^25^ it is unclear which word properties, if any, might be informative in classifying patients with nfvPPA, or other forms of FTD, compared to other disorders.

In healthy adults, production order (PO), sometimes referred to as serial recall order, reveals a pattern of increasing lexical and semantic richness and difficulty^26,27^; but whether this link applies in dementia and aphasia is unknown. Distinct patterns across diagnostic groups could potentially aid diagnostic differentiation and elucidate neurocognitive systems underlying verbal fluency.

This study tested the hypothesis that, in standard clinical versions of the verbal fluency task, the number of words, their psycholinguistic properties and/or PO effects would differentiate neurodegenerative dementias and aphasias. We explored these features in direct comparisons across a large data set collected from a broad range of patient groups including amnestic presentation of Alzheimer’s disease, behavioural and language variants of FTD and the ‘Parkinson-plus’ disorders of PSP and CBS.

The study had three specific aims: (i) to assess whether the total number of words produced during a verbal fluency task can differentiate between diagnostic groups, having controlled for individual differences in age, gender and education; (ii) to identify the multivariate lexico-semantic features of words generated by patients, using a principal component analysis (PCA) to reduce dimensionality; and (iii) to determine the association between the item-level lexico-semantic features and their PO, for each diagnostic group.

## Materials and methods

### Participants and data acquisition

We analysed data from people with clinical diagnoses of Alzheimer’s disease (*n* = 18),^28^ behavioural variant of FTD (bvFTD; *n* = 16),^29^ CBS (*n* = 17),^30^ PSP (*n* = 36),^31^ nfvPPA (*n* = 26) and svPPA (*n* = 26),^32,33^ as well as healthy controls (*n* = 33). Patients were recruited from specialist memory and movement disorders clinics at Cambridge University Hospitals NHS Trust in observational studies (REC references 07/Q0102/3, 10/H0308/34, 12/EE/0475, 14/LO/2045 and 16/LO/1735).

Verbal fluency tests were administered during the baseline visit. Participants were asked to name as many words as they could that (i) began with the letter ‘P’ (excluding people and place names) and (ii) that belonged to the category of ‘animals’, to assess phonemic/letter and semantic/category fluency, respectively. Words were recorded over 60 s for each task and transcribed by the examiner. Features (e.g. frequency and concreteness) were extracted for bigrams when available; otherwise, the bigram was included as a single entry. Where ratings for pluralized words were unavailable, the word properties for the singular version were extracted. In case of homonyms, when the intended word was unclear, the most frequent word was transcribed then analysed. We indicated ‘NA’ for features for which a rating was not available. Where a feature was not available for a given word (i.e. outside of the psycholinguistic databases), the word was excluded from the PCA. The total word count, excluding errors and repetitions, was calculated.

For each of the words, we obtained ratings for psycholinguistic properties from the MRC Psycholinguistic Database^34^ and the English Lexicon Project^35^ as listed in Table 1.

**Table 1:** Word properties and definitions

Word property	Definition
Frequency	How many times a word appears in a corpus
Imageability	How well a word gives rise to a sensory experience or mental image (e.g. ‘pots’ is more imageable than ‘possibility’)
Age of acquisition	When individuals typically learn a word in spoken or written form (e.g. ‘pterodactyl’ is acquired at a later age than ‘people’)
Familiarity	The degree to which an individual comes in contact with or thinks about the concept
Concreteness	The degree to which a word is considered more concrete or more abstract (e.g. the concept of ‘elephants’ is considered highly concrete, whereas ‘paradox’ is abstract)
Length	Number of letters in a word
Orthographic Levenshtein distance (OLD)	The number of insertions, deletions and substitutions needed to generate 1 letter string from another; the mean orthographic distance to the 20 closest orthographic neighbours (e.g. a word with low orthographic neighbourhood like ‘veil’ will have a higher OLD value based on the number of changes needed to create words like ‘boil’, ‘cell’ and ‘fell’ than a word with high orthographic neighbourhood like ‘lime’ to create words like ‘lie’, ‘dime’ and ‘limb’)
Phonological Levenshtein distance (PLD)	The mean number of steps required through phonemic substitutions, insertions or deletions to transform a word into its 20 closest phonological neighbours (e.g. a word with a distant phonological neighbourhood like ‘insomnia’ will have a high PLD based on the number of changes needed to create words like ‘insignia,’ ‘inertia’ and ‘anaemia’ relative to a word like ‘resume’ to create words like ‘result,’ ‘refute’ and ‘legume’)
Semantic neighbourhood density (SND)	The proximity of semantic neighbours to a target word (e.g. an animal such as ‘dog’ has a low semantic distance with a dense neighbourhood since its nearest neighbours such as a ‘cat’ would be relatively close to the target word, as compared to an animal like a ‘kangaroo’)
Semantic diversity	The degree to which the different contexts associated with a given word vary in their meanings (e.g. words such as ‘place’ or ‘people’ have higher values as they appear in more diverse context than words like ‘peacock’)

### Statistical analysis

Between-group differences for total word count were tested by two-way analysis of covariance (ANCOVA) with fluency type (i.e. letter and category) as a within-subject factor, and age and sex as covariates. To compare the levels of impairment on category relative to letter fluency, *Z*-scores were computed to indicate each patient’s category and letter fluency scores in standard deviations from the mean of the healthy control performance. Between-group differences for these *Z*-scores were tested by two-way ANCOVA with fluency type as a within-subject factor, and age and sex as covariates. All *post hoc* analyses were conducted using Tukey’s honestly significant difference (HSD) for multiple comparisons.

To establish whether verbal fluency can indicate not only the presence, but also the severity of global cognitive impairment, we computed correlations across participants to assess two forms of associations: (i) between Addenbrooke’s Cognitive Examination Revised (ACE-R) and total word count, plus word counts for letter and category fluency separately, and (ii) between letter and category fluency.

Next, average ratings per participant for the 10-word properties listed in Table 1 for both fluency tasks combined were entered into a PCA. A Kaiser–Meyer–Olkin test determined the suitability of our data set for PCA. We selected three components based on Cattell’s criteria and then performed varimax rotation. Using factor scores per participant, we conducted a one-way analysis of variance (ANOVA) to test for group differences followed by *post hoc* analyses using Tukey’s HSD for multiple comparisons.

For item-level ‘production order–psycholinguistic feature’ scoring, each word was scored according to its PO position and the three psycholinguistic features that were individually most strongly associated with principal components 1–3—namely: length, imageability and word frequency. For each diagnostic group and over the whole study population, correlation coefficients (Spearman’s rho) were calculated between the PO and these three psycholinguistic features. To capture individual variability of strength and direction, linear regression analyses were run to assess the relationship between the PO and the aforementioned features for each participant. Beta coefficients were extracted to test within- and between-group differences via a six group × two fluency type ANOVA.

Lastly, logistic regression analyses were conducted to ascertain which measure could best discriminate different patient groups and controls. Receiver operating characteristic (ROC) curves were generated using the pROC package.^36^ Age and sex were included as covariates. All statistical analyses were performed in R statistical software.

## Results

### Demographics

Demographic details are shown in Table 2. Ethnicity data were not available for 22 participants. Of the remaining 150 participants, all were ‘White’ (149 ‘White British’ and 1 ‘White-Other’). This demographic is consistent with the office of national statistics 2019 census data which reports that for the East of England, where the majority of our patients live(d), 97% of those over age of 65 are ‘White’.

**Table 2:** Demographics for the study population recorded at the baseline visit

	Control	AD	bvFTD	svPPA	nfvPPA	CBS	PSP	*P*-value*
*N*	33	18	16	26	26	17	36	—
Mean age (years)	68	67	60	66	71	72	72	<0.0001
Sex/gender (female/male)	14/19	10/8	9/7	9/16	14/12	13/4	15/21	0.15
Handedness (right/left)	33/1	11/2	12/2	14/3	21/1	12/0	27/5	0.34
Mean age at leaving full-time education (years)	18	17	17	18	16	17	17	0.07
Mean ACE-R (/100)	96	64	71	64	75	70	78	<0.0001
Mean MMSE (/30)	29	21	25	26	25	23	26	<0.0001

ACE-R, Addenbrooke’s Cognitive Examination Revised; MMSE, Mini-Mental State Examination. *P-value for *F*-test of group difference by ANOVA.

Pairwise multiple comparisons confirmed that, as expected, patients with bvFTD were younger than those with CBS (*P* < 0.001), nfvPPA (*P* < 0.001), PSP (*P* < 0.001) and controls (*P* = 0.02). No other groups differed in age. There were no significant differences between groups in terms of baseline education, handedness or gender. There were differences between groups on the ACE-R and Mini-Mental State Examination (MMSE), with controls performing better than all patient groups on both tests. Patients with PSP scored higher on the ACE-R than those with svPPA (*P* = 0.02) and Alzheimer’s disease (*P* = 0.02). Patients with Alzheimer’s disease scored lower on the MMSE than those with svPPA (*P* = 0.01), nfvPPA (*P* = 0.05) and PSP (*P* = 0.004).

### Diagnostic differentiation

#### Total word count

**Figure 1:**
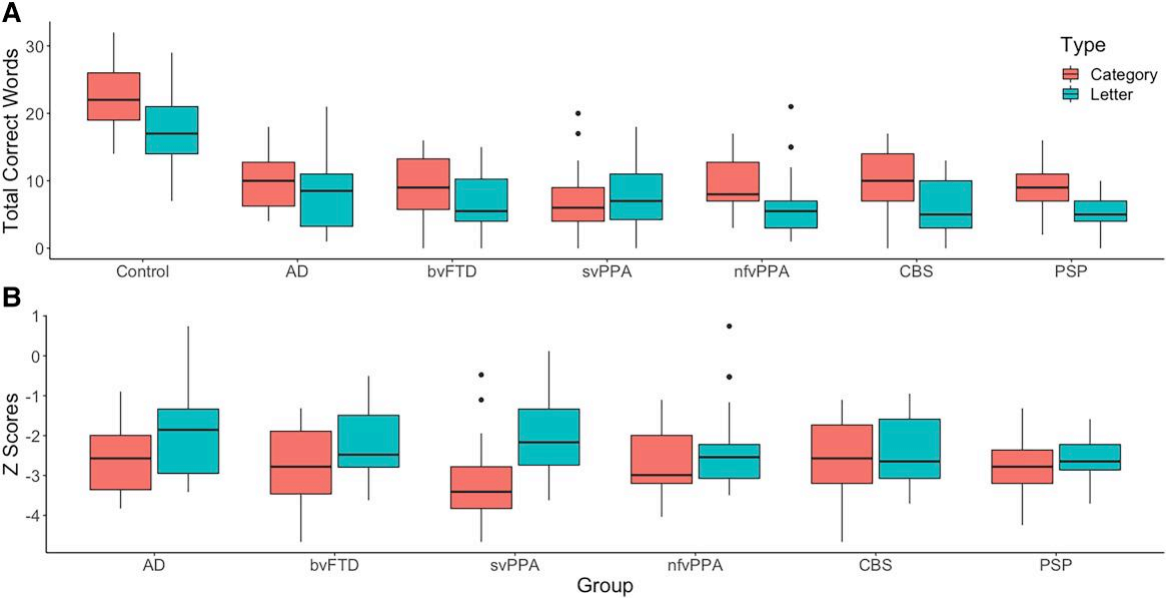
**Total words and *Z*-scores for category and letter fluency.** (**A**) Total words produced by controls and patient groups during category and letter fluency. ANCOVA revealed a main effect of group [*F*(6324) = 43.4, *P* < 0.001] and a group-by-fluency type interaction [*F*(6324) = 2.4, *P* = 0.03]. Group data illustrate healthy controls performing (i) better than all patient groups and (ii) better on category relative to letter fluency. (**B**) *Z*-scores for category and letter fluency exhibited by patient groups. ANCOVA revealed an effect of group [*F*(5260) = 2.7, *P* = 0.02], fluency type [*F*(1260) = 4.8, *P* = 0.03] and a group-by-fluency type interaction [*F*(5260) = 2.5, *P* = 0.04]. *Post hoc* analyses revealed a significant difference in letter versus category *Z*-scores in svPPAs (*P* < 0.001) only.

As shown in Fig. 1A, there was a main effect of group on the total word count, in both letter and category tasks, after controlling for age and sex. Specifically, results of an ANCOVA revealed an effect of group [*F*(6324) = 43.4, *P* < 0.001], indicating that controls produced more words than all patient groups (control versus each patient group, *P* < 0.001), and group-by-fluency type interaction [*F*(6324) = 2.4, *P* = 0.03]. *Post hoc* analyses with Tukey’s HSD multiple comparisons correction revealed a significant difference between the total word count in letter versus category fluency in controls (*P* < 0.001) and (marginally significant) in PSP (*P* = 0.07).

To compare the levels of impairment on category relative to letter fluency, *Z*-scores were computed to indicate each patient’s category and letter fluency scores in standard deviations from the mean of the healthy control performance (see Fig. 1B). After adjustment of age and sex, results from an ANCOVA revealed an effect of group [*F*(5260) = 2.7, *P* = 0.02], fluency type [*F*(1260) = 4.8, *P* = 0.03] and a group-by-fluency type interaction [*F*(5260) = 2.5, *P* = 0.04]. *Post hoc* analyses with Tukey’s HSD multiple comparisons correction revealed a significant difference between the *Z*-scores in letter versus category fluency in svPPAs (*P* < 0.001) only.

#### Associations between global severity, letter and category fluency

**Figure 2:**
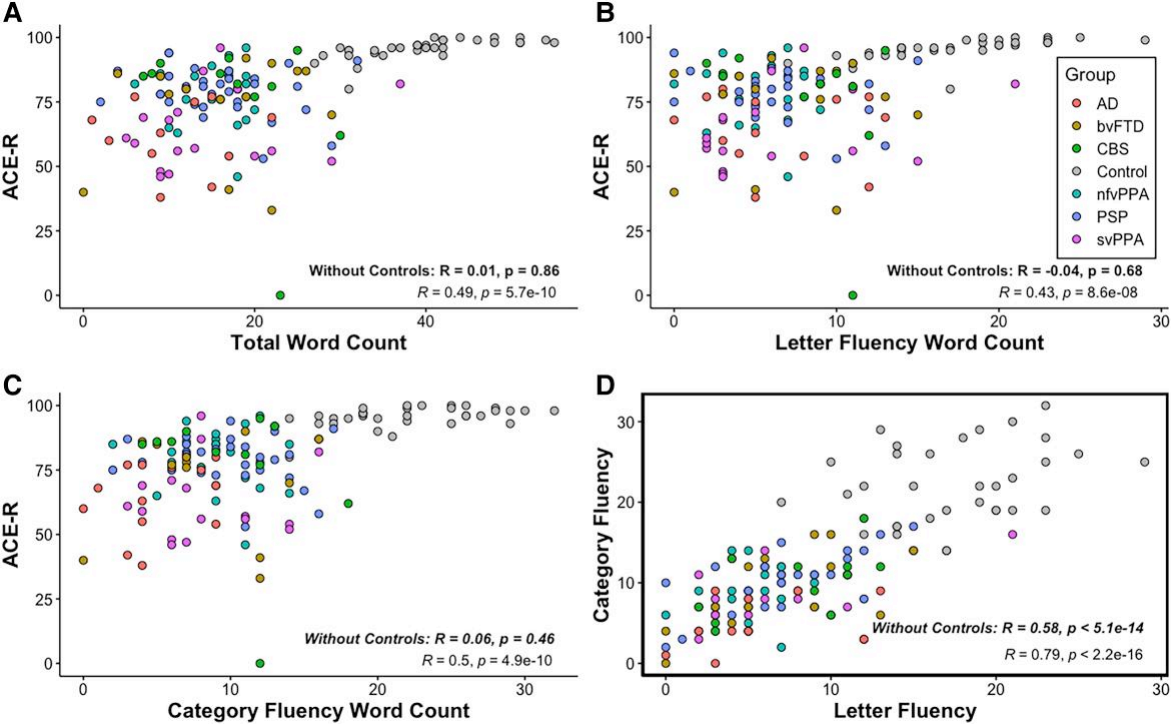
**Associations between ACE-R and word counts and between letter and category fluency.** (**A**) ACE-R was positively correlated with total word count, *r*(138) = 0.49, *P* < 0.001. (**B**) ACE-R was positively correlated with letter fluency word count, *r*(138) = 0.43, *P* < 0.001. (**C**) ACE-R was positively correlated with category fluency word count, *r*(138) = 0.50, *P* < 0.001. (**D**) Letter fluency word count was positively correlated with category fluency word count, *r*(138) = 0.79, *P* < 0.001. Correlations without healthy controls are indicated in bold.

A Pearson correlation coefficient was computed to assess the linear relationship between (i) ACE-R, as a measure of global severity for patients, and total word count, word count for letter and category fluency, and (ii) word count for letter versus category fluency. As shown in Fig. 2, ACE-R was positively correlated with total word count, *r*(138) = 0.49, *P* < 0.001, letter fluency word count, *r*(138) = 0.43, *P* < 0.001, and category fluency word count, *r*(138) = 0.50, *P* < 0.001. A positive correlation was also found between letter and category fluency, *r*(138) = 0.79, *P* < 0.001. When healthy controls were excluded, very weak correlations were found between ACE-R and (i) total word count, *r*(134) = 0.01, *P* = 0.86, (ii) letter fluency word count, *r*(134) = −0.04, *P* = 0.68, and (iii) category fluency word count, *r*(134) = 0.06, *P* = 0.46. A positive correlation was found between letter and category fluency, *r*(137) = 0.58, *P* < 0.001.

#### Syndromic dimensions of word properties using principal component analysis

Mean ratings for each word property per participant, excluding controls, were entered into a PCA with varimax rotation. Three principal components were identified using Cattell’s criteria, each representing a group of covarying psycholinguistic features of words produced by patients. Three components explained 87.3% of the variance (Kaiser–Meyer–Olkin = 0.77). The loading of each measure is given in Table 3.

**Table 3:** Loadings for PCA of word properties

Measure	PC 1 (phonological length)	PC 2 (semantic richness)	PC 3 (lexical familiarity)
Length	**0**.**91**	−0.24	−0.28
OLD	**0**.**93**	0.00	−0.33
PLD	**0**.**93**	0.00	−0.29
Concreteness	0.00	**0**.**95**	0.00
Imageability	0.00	**0**.**96**	0.00
Age of acquisition	0.38	**−0**.**66**	**−0**.**55**
Semantic diversity	0.13	**−0**.**60**	**0**.**50**
Frequency	**−0**.**50**	0.00	**0**.**78**
Familiarity	−0.36	0.20	**0**.**78**
SND	−0.49	−0.13	**0**.**77**

Rotation: orthogonal varimax. Loadings above a threshold of 0.5 are in bold. PC, principal component; OLD, orthographic Levenshtein distance; PLD, phonological Levenshtein distance; SND, semantic neighbourhood density.

**Figure 3:**
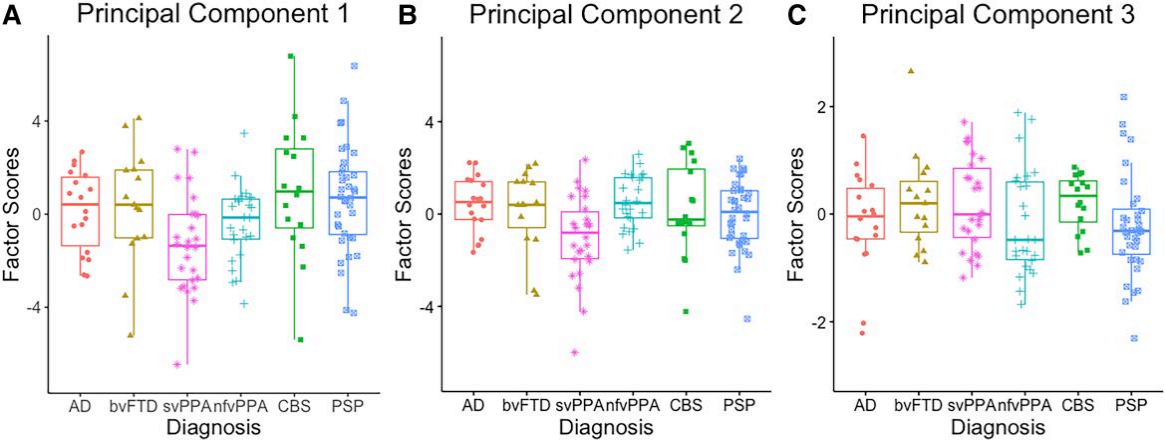
**Principal component analysis scores of word properties across diagnostic groups.** (**A**) PC 1: ‘phonological length’; a one-way ANOVA revealed group differences in PC 1 [*F*(5131) = 3.38, *P* = 0.007]. (**B**) PC 2: ‘semantic richness’; a one-way ANOVA revealed group differences in PC 2 [*F*(5131) = 3.20, *P* = 0.009]. (**C**) PC 3: ‘lexical familiarity’; a one-way ANOVA did not reveal group differences in PC 3 [*F*(5131) = 0.300 *P* = 0.42]. AD, Alzheimer’s disease; bvFTD, behavioural variant frontotemporal dementia; svPPA, semantic variant primary progressive aphasia; nfvPPA, non-fluent variant primary progressive aphasia; CBS, corticobasal syndrome; PSP, progressive supranuclear palsy.

Principal component (PC) 1 (see Fig. 3A) was loaded heavily by length, orthographic Levenshtein distance (OLD) and phonological Levenshtein distance (PLD), and was thus interpreted as ‘phonological length’ where positive scores reflect a greater production of words that are longer (e.g. ‘prescription’ and ‘psychological’ versus ‘pan’ and ‘pen’) with high phonological and orthographic Levenshtein distance (e.g. ‘plum’, ‘premature’ and ‘proliferate’ versus ‘pen’, ‘pan’ and ‘pat’). The results from a one-way ANOVA revealed group differences in PC 1 [*F*(5131) = 3.38, *P* = 0.007], driven by patients with svPPA producing shorter and phonologically and orthographically less complex words than patients with PSP (*P* = 0.008) and CBS (*P* = 0.02).

Principal component 2 (Fig. 3B) was interpreted as ‘semantic richness’ since concreteness, imageability, age of acquisition and semantic diversity were loaded heavily on it. Positive scores represented a greater production of highly imageable and concrete words that are acquired earlier and have less semantically diverse meanings (e.g. ‘cow’ and ‘elephant’ versus ‘fowl’ and ‘louse’). Group differences were found for PC 2 [*F*(5131) = 3.20, *P* = 0.009], driven by patients with svPPA producing less concrete, less imageable, more semantically diverse and later acquired words relative to patients with nfvPPA (*P* = 0.005) and Alzheimer’s disease (*P* = 0.04).

Principal component 3 (Fig. 3C) was loaded heavily by lexico-semantic features including frequency, familiarity and semantic neighbourhood density and was interpreted as ‘lexical familiarity’. Positive scores represented a greater production of more frequent and familiar words with higher semantic density (e.g. ‘dog’ and ‘fish’ versus ‘tiger’ and ‘snake’). A one-way ANOVA did not reveal group differences for PC 3 [*F*(5131) = 0.300 *P* = 0.42].

#### Item-level fluency with production order

**Figure 4:**
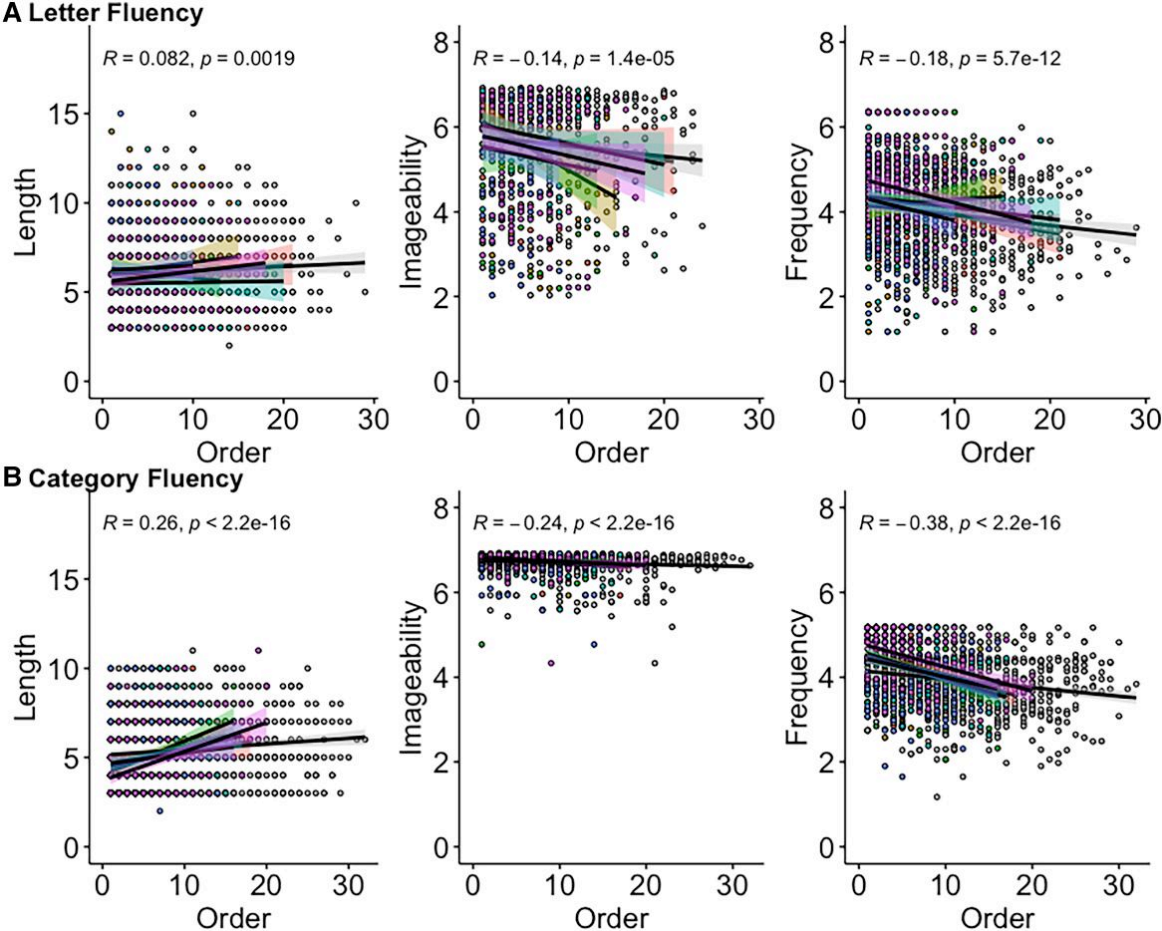
**Production order (PO)-word feature scatterplots over the whole study population**. (**A**) PO-length (left), PO-imageability (middle) and PO-frequency (right) for letter fluency. (**B**) PO-length (left), PO-imageability (middle) and PO-frequency (right) for category fluency. The *x*-axis represents production order, and the *y*-axis shows the length count, as well as ratings for imageability and frequency.

Correlation coefficients (Spearman’s rho) were calculated for each participant between the PO and the three psycholinguistic features that were individually most strongly associated with principal components 1–3, namely length, imageability and frequency. Figure 4 plots the ‘PO-psycholinguistic feature’ trends averaged across groups with PO in the *x*-axis and the ratings of length, imageability and frequency of the words produced in the *y*-axis. The ‘PO-length’ effect was positive for letter (*P* = 0.002) and category (*P* < 0.001) over the whole study population and for each group except letter fluency in CBS (*r* = −0.06), although CBS had the most positive correlation for category fluency (*r* = 0.46). The ‘PO-imageability’ effect was negative for letter (*P* < 0.001) and category (*P* < 0.001) over the whole study population and for each group. The most negative correlations were found in bvFTD for letter fluency (*r* = −0.39) and nfvPPA and svPPA for category fluency (*r* = −0.31). The ‘PO-frequency’ effect was negative for both letter (*P* < 0.001) and category (*P* < 0.001) over the whole study population and for each group except letter fluency in CBS (*r* = 0.03) and bvFTD (*r* = 0.03). Patients with svPPA showed the most negative correlation during category fluency (*r* = −0.44). Correlation coefficients for all psycholinguistic features and PO can be found in Supplementary Table 1.

Linear regression analyses were used to assess the relationship between the PO and length, imageability and frequency for each participant, and beta coefficients were extracted to test within- and between-group differences. Six groups × two fluency type ANOVAs failed to reveal any effect of group or type for length [group: *F*(6314) = 1.34, *P* = 0.24; type: *F*(1314) = 2.28, *P* = 0.13], imageability [group: *F*(6299) = 1.34, *P* = 0.24; type: *F*(1299 = 1.53, *P* = 0.87] or frequency [group: *F*(6315) = 1.72, *P* = 0.12; type: *F*(1315) = 2.82, *P* = 0.09].

#### Logistic regression

**Figure 5:**
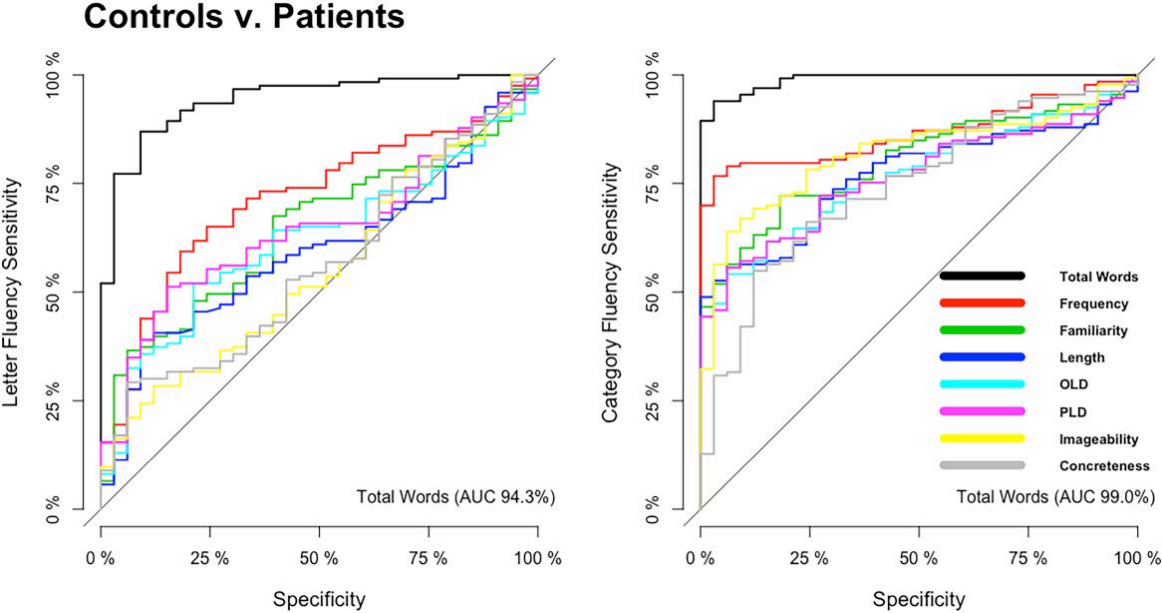
Receiver operating characteristic curves distinguishing between controls versus all patient groups.

**Figure 6:**
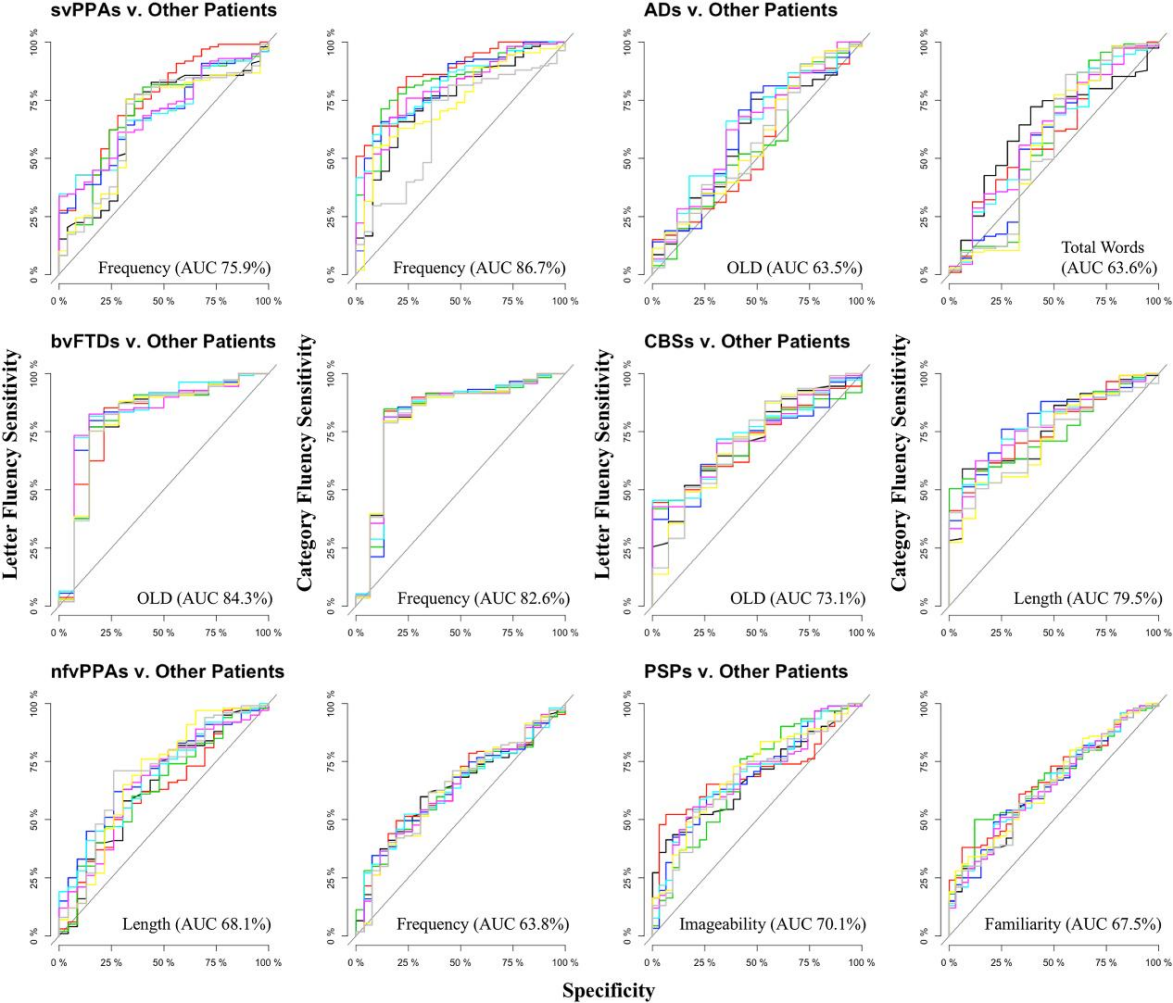
Receiver operating characteristic curves distinguishing each patient group against other patient groups.

Logistic regression analyses were conducted to determine which measures (i.e. total word count and/or the psycholinguistic properties associated with these words) could discriminate (i) controls versus all patient groups and (ii) between one patient group and the others after adjusting for age and sex. The discrimination between groups is reported as ROC curves in Figs 5 and 6.

As shown in Fig. 5, when comparing controls relative to all patients, the strongest discriminator for letter fluency was total word count (AUC = 94.3%) followed by word frequency (AUC = 71.6%). For category fluency, the strongest discriminators were also total word count (AUC = 99.0%) and word frequency (AUC = 86.7%).

The fluency metrics did not discriminate between most patient groups with high accuracy (see Fig. 6). A partial exception was the moderate discrimination of (i) svPPA against all other patient groups with word frequency as the strongest discriminator for letter (AUC = 75.9%) and category (AUC = 86.7%) fluency and (ii) CBS against all other patient groups with OLD (AUC = 84.3%) and frequency (AUC = 82.6%) as the strongest discriminators for letter and category fluency, respectively. The discrimination of each of the other patient groups was weak, at best. Orthographic Levenshtein distance (AUC = 63.5%) and total word count (AUC = 63.6%) were the best measures for Alzheimer’s disease patients in letter and category fluency, respectively. For CBS patients, OLD (AUC = 73.1%) and length (AUC = 79.5%) were the best measures for letter and category fluency. Length (AUC = 68.1%) and frequency (AUC = 63.8%) were the best measures for letter and category fluency for nfvPPA patients. Imageability (AUC = 70.1%) and familiarity (AUC = 67.5%) were the best measures for letter and category fluency for PSP patients.

## Discussion

Although verbal fluency tests are one of the most popular assessments regularly administered in clinic, how well they can differentiate either all patient types from healthy controls or between patient groups has not previously been established. Furthermore, there has been little or no exploration in previous studies of whether differential diagnosis between various forms of cortical and subcortical neurodegenerative diseases can be improved with quantitative, albeit time-consuming, analyses of properties of the words produced. Using a large data set collected from a broad range of neurodegenerative patient groups, we addressed these issues by examining letter and category fluency assessed at the first clinical visit using quantification of total word count and analysis of the qualities of the words produced. There were two very clear principal results: (i) the number of words produced in letter and/or category fluency strongly differentiated healthy controls from each neurodegenerative disease (amnestic Alzheimer’s disease, behavioural and language variants of FTD and both PSP and CBS) and (ii), on the other hand, neither the total word count nor the psycholinguistic properties of the words produced differentiated between disorders, with the partial exception of svPPA (see below). Previous studies of individual patient groups have also reported that the total number of words produced can differentiate healthy controls from those with major neurocognitive disorders,^37^ as well as those with mild cognitive impairment from advanced dementias.^38^ It seems very likely that this lack of diagnostic differentiation derives from the fact that verbal fluency taxes multiple aspects of higher cognition and language. Thus, if any aspect of language, memory, attention or executive functioning is impaired, then performance on verbal fluency will be compromised regardless of specific diagnosis.

Going beyond the traditional measure of the number of words produced, we examined the psycholinguistic characteristics of the words produced by each patient group. These additional psycholinguistic measures showed weak differences between diagnostic groups (Fig. 6). The only partial exception is that word frequency was a moderately strong discriminator in letter (AUC = 75.9%) and category (AUC = 86.7%) fluency for patients with svPPA. These cases were more likely than the other groups to generate items with higher word frequency, which aligns with the shift of word frequency observed in svPPA naming and connected speech.^15–17^ Beyond this moderate effect, our results indicate that only subtle (non-significant), graded differences in lexico-semantic features are found at the group level and are unlikely to provide diagnostic differentiation for individual patients. From a clinical perspective, it is also worth noting that examining the psycholinguistic properties of the words produced by each patient is laborious and would seem to have little clinical utility in light of the subtle differences between the diagnostic groups.

Consistent with prior literature, Table 3 shows the overlapping nature of certain psycholinguistic properties such as frequency and age of acquisition and semantic diversity and concreteness.^39–43^ Word frequency was not only linked to phonological output, namely one’s ability to produce phonologically and orthographically longer words, but also lexical characteristics such as age of acquisition. Similarly, age of acquisition and semantic diversity were loaded heavily on both ‘semantic richness’ (PC 2) and ‘lexical familiarity’ (PC 3). This is in line with previous word production models where psycholinguistic properties interact at different linguistic levels, instead of individual language components, with both feedforward and feedback activations.^44,45^ In particular, word length, semantic diversity and phonological neighbourhood density have been shown to predict greater word production,^39,41,46–50^ whereas frequency and age of acquisition demonstrated robust effects in naming.^17^ In accordance with previous reports of svPPA patients’ increasing restriction to words that are more frequent, more abstract and of high semantic diversity, it is not surprising that svPPA was the only group that was differentiated from frontal lobe syndromes such as nfvPPA and PSP in terms of ‘phonological length’ (PC 1) and ‘semantic richness’ (PC 2).

In addition to the total number of words produced, the type of verbal fluency has often been proposed to differentiate between different kinds of neurodegenerative disorder. Previous studies have reported either equally impaired performance on both types of fluency or better performance on category than letter fluency in patients with FTD, PSP and CBS.^9,12,51^ However, reports of unequal performance as a function of fluency type might merely reflect the normative pattern found in healthy controls.^52^ A reverse of this pattern has been reported in patients with Alzheimer’s disease and SD,^13,53,54^ and thus, it has been proposed that disorders that disrupt semantic memory will result in a more pronounced deficit for category relative to letter fluency.^9^ This phenomenon is thought to arise from a reduction in the availability of semantic attributes following temporal lobe degeneration.^55–58^ Going beyond the raw difference between letter and category fluency, where even healthy controls perform better on category than letter fluency^52,59^ (Fig. 1A), we computed *Z*-scores to indicate each patient’s performance on letter versus category fluency from the mean of the healthy control performance and found a disproportionate category fluency impairment in svPPA patients (Fig. 1B). This disparity has been reported and replicated numerous times in previous studies^54,56,57,60^ and reinforces the idea that temporal lobe degeneration disproportionately affects category fluency performance. Even though the investigation of differential performance in fluency aids diagnostic differentiation for svPPA patients, no other groups showed a similar or opposite (i.e. a disproportionate impairment on letter relative to category fluency) pattern. Given that the multiple patient groups included in our study have characteristic differential anatomical distributions and associated variations in cognitive–language profiles, then we might have expected to observe contrastive effects of fluency type across the groups. Our results, however, only revealed significantly more words produced in category than letter fluency in healthy controls and (marginally) in patients with PSP (Fig. 1A). Indeed, the total numbers of words produced in each type of fluency were highly correlated (Fig. 2D) indicating that they primarily rely on the same cognitive and language processes and to very similar degrees. Thus, our findings question the clinical utility of discrepancy between category and letter fluency for diagnostic differentiation transdiagnostically with the exception temporal lobe predominant syndromes such as svPPA.

There were some limitations to our study. First, we only present clinical, not pathological, diagnoses. Future studies of performance on fluency tasks might explore whether performance relates in any way to the type of pathology as well as the clinical diagnosis. Secondly, we did not directly explore the atrophy correlates of fluency performance. Future work could investigate the relationship between fluency performance and the level/distribution of atrophy in each patient group and in the clinical population as a whole. On the other hand, perhaps the more striking observation is that—despite considerable variations in the types of neurodegenerative patient groups included in our study—there was so little evidence of substantial variations in the number or the pattern of words elicited. This global result suggests that, instead of fluency performance having clear and restricted atrophy correlates, multiple brain systems including cortical and subcortical regions are engaged by tests of verbal fluency. Although prior lesion and functional imaging studies have proposed that distinct brain areas support verbal fluency (e.g. prefrontal executive regions), even brief cognitive deconstruction of the fluency task implicates numerous language, memory and executive systems in good performance. As a result, it is perhaps unsurprising that the key result from this study is that verbal fluency is an excellent, efficient clinical task for assessing the presence and level of global brain–cognitive decline (i.e. differentiates patients from controls) but is very limited in its utility to differentiate between cognitively and anatomically disparate patient groups.

## Conclusion

Our results have important clinical and research implications. The study supports previous claims that verbal fluency tests are clinically efficient and sensitive for detecting cognitive changes across many different types of neurodegenerative condition. In contrast, there was very limited evidence that fluency performance (e.g. total word count) can assist differential diagnosis. Indeed, even detailed investigation of word properties and order of production did not improve diagnostic differentiation, and such analyses are time consuming and impractical in clinical settings.

## Supplementary Material

fcad042_Supplementary_Data

## Data Availability

The authors confirm that the data supporting the findings of this study are available within the article and its Supplementary material. Additional data are available from the corresponding author upon reasonable request.

## Abbreviations

ACE-R =: Addenbrooke’s Cognitive Examination Revised
bvFTD =: behavioural variant frontotemporal dementia
CBS =: corticobasal syndrome
MMSE =: Mini-Mental State Examination
nfvPPA =: non-fluent variant primary progressive aphasia
OLD =: orthographic Levenshtein distance
PCA =: principal component analysis
PLD =: phonological Levenshtein distance
PO =: production order
PSP =: progressive supranuclear palsy
SND =: semantic neighbourhood density
svPPA =: semantic variant primary progressive aphasia
