# Supplementary material for: Verbal fluency tests assess global cognitive status but have limited diagnostic differentiation: evidence from a large-scale examination of six neurodegenerative diseases
Source: Brain Commun. 2023 Feb 21;5(2):fcad042. doi: 10.1093/braincomms/fcad042 (PMC9999359; doi:10.1093/braincomms/fcad042)
Supplement: fcad042_Supplementary_Data [file fcad042_supplementary_data.pdf]

Supplementary material

Supplementary Table 1 Production Order (PO) and all word features

	PO-Len	PO-Imag	PO-Freq	PO-OLD	PO-PLD	PO-Conc	PO-AoA	PO-SD	PO-Fam	PO-SND	Type
Control	r = 0.07, *p* = 0.11	r = -0.15, *p* = 0.009	r = -0.15, *p* < 0.001	r = 0.006, *p* = 0.88	r = 0.02, *p* = 0.58	r = -0.18, *p* = 0.001	r = 0.19, *p* < 0.001	r = -0.07, *p* = 0.13	r = -0.11, *p* = 0.05	r = -0.12, *p* = 0.005	Letter
	r = 0.14, *p* < 0.001	r = -0.18, *p* < 0.001	r = -0.23, *p* < 0.001	r = 0.12, *p* = 0.003	r = 0.10, *p* = 0.01	r = -0.12, *p* = 0.003	r = 0.20, *p* < 0.001	r = -0.03, *p* = 0.45	r = -0.17, *p* < 0.001	r = -0.20, *p* < 0.001	Category
AD	r = 0.17, *p* = 0.05	r = -0.16, *p* = 0.11	r = -0.26, *p* = 0.002	r = 0.19, *p* = 0.03	r = 0.18, *p* = 0.04	r = -0.22, *p* = 0.02	r = 0.32, *p* = 0.001	r = -0.01, *p* = 0.88	r = -0.26, *p* = 0.008	r = -0.14, *p* = 0.11	Letter
	r = 0.22, *p* = 0.004	r = -0.29, *p* = 0.002	r = -0.38, *p* < 0.001	r = 0.19, *p* = 0.01	r = 0.16, *p* = 0.04	r = -0.12, *p* = 0.15	r = 0.36, *p* < 0.001	r = 0.04, *p* = 0.64	r = -0.34, *p* < 0.001	r = -0.32, *p* < 0.001	Category
bvFTD	r = 0.13, *p* = 0.20	r = -0.39, *p* < 0.001	r = 0.03, *p* = 0.78	r = 0.14, *p* = 0.17	r = 0.17, *p* = 0.08	r = -0.39, *p* < 0.001	r = 0.28, *p* = 0.02	r = 0.13, *p* = 0.18	r = -0.27, *p* = 0.03	r = 0.08, *p* = 0.39	Letter
	r = 0.28, *p* = 0.001	r = -0.16, *p* = 0.06	r = -0.34, *p* < 0.001	r = 0.25, *p* = 0.003	r = 0.21, *p* = 0.01	r = -0.09, *p* = 0.29	r = 0.26, *p* = 0.002	r = -0.02, *p* = 0.82	r = -0.34, *p* < 0.001	r = -0.39, *p* < 0.001	Category
svPPA	r = 0.10, *p* = 0.18	r = -0.18, *p* = 0.04	r = -0.23, *p* = 0.001	r = 0.06, *p* = 0.41	r = 0.09, *p* = 0.19	r = -0.21, *p* = 0.02	r = 0.28, *p* < 0.001	r = -0.07, *p* = 0.31	r = -0.31, *p* < 0.001	r = -0.18, *p* = 0.01	Letter
	r = 0.36, *p* < 0.001	r = -0.31, *p* < 0.001	r = -0.44, *p* < 0.001	r = 0.28, *p* < 0.001	r = 0.18, *p* = 0.02	r = -0.02, *p* = 0.83	r = 0.49, *p* < 0.001	r = 0.07, *p* = 0.38	r = -0.43, *p* < 0.001	r = -0.35, *p* < 0.001	Category
nfvPPA	r = 0.09, *p* = 0.25	r = -0.15, *p* = 0.13	r = -0.15, *p* = 0.05	r = 0.08, *p* = 0.31	r = 0.07, *p* = 0.41	r = -0.17, *p* = 0.10	r = 0.14, *p* = 0.17	r = -0.10, *p* = 0.24	r = -0.08, *p* = 0.43	r = -0.09, *p* = 0.24	Letter
	r = 0.29, *p* < 0.001	r = -0.31, *p* < 0.001	r = -0.42, *p* < 0.001	r = 0.29, *p* < 0.001	r = 0.23, *p* < 0.001	r = -0.10, *p* = 0.14	r = 0.42, *p* < 0.001	r = -0.05, *p* = 0.48	r = -0.38, *p* < 0.001	r = -0.35, *p* < 0.001	Category

CBS	r = -0.07, *p* = 0.55	r = -0.09, *p* = 0.47	r = 0.03, *p* = 0.78	r = -0.11, *p* = 0.29	r = -0.05, *p* = 0.66	r = -0.14, *p* = 0.29	r = -0.06, *p* = 0.65	r = -0.02, *p* = 0.82	r = -0.16, *p* = 0.22	r = -0.03, *p* = 0.75	Letter
	r = 0.46, *p* < 0.001	r = -0.21, *p* = 0.01	r = -0.39, *p* < 0.001	r = 0.44, *p* < 0.001	r = 0.35, *p* < 0.001	r = -0.12, *p* = 0.15	r = 0.38, *p* < 0.001	r = 0.12, *p* = 0.16	r = -0.34, *p* < 0.001	r = -0.39, *p* < 0.001	Category
PSP	r = 0.03, *p* = 0.73	r = -0.05, *p* = 0.57	r = -0.10, *p* = 0.16	r = 0.05, *p* = 0.50	r = 0.08, *p* = 0.29	r = -0.12, *p* = 0.19	r = 0.10, *p* = 0.25	r = -0.12, *p* = 0.11	r = -0.13, *p* = 0.16	r = -0.14, *p* = 0.06	Letter
	r = 0.16, *p* = 0.007	r = -0.12, *p* = 0.04	r = -0.26, *p* < 0.001	r = 0.12, *p* = 0.04	r = 0.06, *p* = 0.29	r = -0.05, *p* = 0.36	r = 0.25, *p* < 0.001	r = -0.04, *p* = 0.52	r = -0.22, *p* < 0.001	r = -0.21, *p* < 0.001	Category

AD, Alzheimer's disease; AoA, age of acquisition; bvFTD, behavioural variant frontotemporal dementia; CBS, corticobasal syndrome; Conc, concreteness; Fam, familiarity; Freq, frequency; Imag, imageability; Len, length; nfvPPA, non-fluent variant primary progressive aphasia; OLD, orthographic levenshtein distance; PLD, phonological levenshtein distance; PSP, progressive supranuclear palsy; SD, semantic diversity; SND, semantic neighbourhood density; svPPA, semantic variant primary progressive aphasia.
